# A Multiplex One-Tube Nested Real-Time PCR Assay for the Point-of-Care Testing of Infectious Meningitis

**DOI:** 10.3390/pathogens15050456

**Published:** 2026-04-22

**Authors:** Duoxiao Zhang, Jie Wang, Zijin Zhao, Yanqing Tie, Jianing Wu, Shihao Jiao, Xingyu Liu, Yuxin Wang, Shijue Gao, Mengchuan Zhao, Pei Zhao, Zhiqiang Han, Xiaona Lyu, Xinxin Shen, Xuejun Ma, Zhishan Feng

**Affiliations:** 1 Graduate School, Hebei North University, Zhangjiakou 075000, China; zhangduoxiao99@163.com (D.Z.); 18731157791@163.com (J.W.); 2 Department of Clinical Laboratory, Hebei General Hospital, Shijiazhuang 050051, China; wangjie060110@163.com (J.W.); zhaozijin1@foxmail.com (Z.Z.); tyq1995@126.com (Y.T.); jiaosh0203@163.com (S.J.); ysl0517@126.com (X.L.); wyx18831995115@126.com (Y.W.); zhaomengchuan1989@163.com (M.Z.); zhaopei21980@163.com (P.Z.); 18731362725@163.com (Z.H.); lvxiaona2020@163.com (X.L.); 3 Hebei Key Laboratory of Molecular Medicine, Shijiazhuang 050051, China; 4 Hebei Clinical Research Center for Laboratory Medicine, Shijiazhuang 050051, China; 5 National Key Laboratory of Intelligent Tracking and Forecasting for Infectious Diseases, NHC Key Laboratory of Medical Virology and Viral Diseases, National Institute for Viral Disease Control and Prevention, Chinese Center for Disease Control and Prevention, Beijing 102206, China; gsj_0318@163.com; 6 Graduate School, North China University of Science and Technology, Tangshan 063210, China; 7 Graduate School, Hebei Medical University, Shijiazhuang 050031, China

**Keywords:** cerebrospinal fluid, *Haemophilus influenzae*, *Listeria monocytogenes*, *Cryptococcus neoformans*, multiplex one-tube nested real-time fluorescent quantitative PCR (mONRT-PCR), multiplex testing, POCT

## Abstract

In this study, we developed a multiplex one-tube nested real-time fluorescent quantitative PCR (mONRT-PCR) assay integrated with a portable, fully automated nucleic acid point-of-care testing (POCT) platform for the detection of *Haemophilus influenzae* (*H. influenzae*), *Listeria monocytogenes* (*L. monocytogenes*), and *Cryptococcus neoformans* (*C. neoformans*) in cerebrospinal fluid (CSF). The assay enables nested amplification within a closed system using conventional primers and probes, thereby reducing operational complexity and minimizing contamination risk. Analytical evaluation demonstrated limits of detection of 10^0^ copies/μL for *H. influenzae* and *L. monocytogenes*, and 10^1^ copies/μL for *C. neoformans* using recombinant plasmids, as well as 10^−7^ to 10^−6^ ng/μL using genomic DNA. No cross-reactivity was observed when tested against a panel of 17 common non-target microorganisms encountered in clinical microbiology laboratories. In simulated CSF samples, the assay maintained detectable amplification at low pathogen concentrations. When implemented on the POCT platform, detection limits reached 5, 10, and 50 CFU/mL for the three pathogens, respectively. Clinical evaluation using 43 CSF samples showed almost perfect agreement with conventional qPCR (κ = 0.861, *p* < 0.001). Notably, additional *C. neoformans* detections were observed by mONRT-PCR-POCT compared with qPCR, suggesting improved sensitivity under clinical conditions. The assay yielded results within approximately 1 h and 47 min. These findings indicate that the proposed assay provides a rapid, sensitive, and integrated approach for meningitis pathogen detection, while maintaining a practical balance between analytical performance and operational simplicity. Further validation in larger cohorts is warranted.

## 1. Introduction

Infectious meningitis is a severe disease characterized by inflammation of the meninges and remains a significant public health issue globally, associated with considerable morbidity and mortality [1,2]. Early and accurate pathogen identification is recognized as a key component in global meningitis control strategies, including initiatives proposed by the World Health Organization [3]. Notably, *Haemophilus influenzae* (*H. influenzae*), *Listeria monocytogenes* (*L. monocytogenes*), and *Cryptococcus neoformans* (*C. neoformans*) are clinically important bacterial and fungal pathogens associated with meningitis. *H. influenzae* is an important bacterial pathogen that causes invasive infections, including meningitis, particularly in pediatric populations. Despite the introduction of *H. influenzae* type b (Hib) conjugate vaccines, *H. influenzae* remains associated with meningitis cases, particularly in settings with limited vaccination coverage [4]. *L. monocytogenes* is a recognized cause of bacterial meningitis, characterized by its ability to invade the central nervous system, posing diagnostic challenges in clinical practice [5,6]. *C. neoformans* is the most common fungal meningitis pathogen, often causing severe central nervous system infections, highlighting the urgent need for rapid, sensitive detection technologies targeting these pathogens [7,8]. These three pathogens were selected to represent distinct microbiological categories involved in meningitis, including Gram-negative bacteria, Gram-positive bacteria, and encapsulated fungi. Bacterial pathogens causing meningitis include both Gram-negative bacteria (e.g., *H. influenzae*) and Gram-positive bacteria (e.g., *L. monocytogenes*). By encompassing organisms with diverse biological characteristics, this study aimed to evaluate the adaptability and robustness of the multiplex one-tube nested PCR platform across different pathogen types within a single closed-tube system. Given the complexity and diversity of meningitis pathogens, as a proof of concept study, the present study focused on a limited number of representative organisms for initial platform validation of the adaptability and robustness. This study therefore represents an initial validation of the platform rather than the development of a comprehensive meningitis diagnostic panel. Other pathogens associated with meningitis include *Streptococcus pneumoniae* and *Neisseria meningitidis*, as well as various viral and fungal agents. These pathogens contribute to the etiological diversity of meningitis across different clinical settings [9].

Currently, laboratory diagnosis of bacterial pathogens in CSF primarily relies on traditional microbiological methods. Routine CSF culture remains the gold standard for diagnosing bacterial meningitis pathogens. However, empirical treatment prior to culture reduces its sensitivity, particularly when patients have received antibiotic therapy before CSF collection, and the process is time-consuming (typically requiring 24–72 h). In addition, previous large epidemiological studies have reported that up to 33% of clinically diagnosed bacterial meningitis cases yielded negative culture results [10]. For fungal meningitis caused by *C. neoformans*, culture-based methods are even more time-consuming (requiring 96–120 h for results) and yield only a 30% positivity rate [11,12]. In addition, latex agglutination tests, colloidal gold immunochromatography, and India ink staining [13] may yield false-negative results for capsular-deficient or acapsular strains and have limited ability to differentiate *C. neoformans* from *Cryptococcus gattii* [14,15,16,17]. To overcome limitations of culture-based methods, various polymerase chain reaction (PCR)-based approaches have been widely adopted for molecular diagnosis of infectious meningitis [1,18]. However, these methods exhibit significant variability in analytical sensitivity and limited validation in prospective studies, raising questions about their practicality for clinical testing. Among commercially available qPCR systems, only one currently holds U.S. approval. The U.S. Food and Drug Administration (FDA)-recommended FilmArray^®^ Meningitis/Encephalitis (ME) Test Panel (FilmArray ME Panel) [19] employs multiplex nested PCR combined with melting curve analysis to detect 14 common pathogens within one hour. However, existing studies primarily focus on positive agreement rates, with limited evaluation of analytical sensitivity and performance in CSF samples with low pathogen loads [20]. Overall, clinically accessible, simple, rapid, and highly sensitive non-culture molecular diagnostic methods for low-pathogen-load CSF specimens remain insufficient. Therefore, there remains a need for rapid molecular assays that can serve as early adjunct diagnostic tools to support clinical decision-making, particularly when pathogen loads are low in the clinical samples or prior antimicrobial therapy has been administered. The intended role of this assay is to provide rapid molecular support in suspected infectious meningitis cases while conventional microbiological results are pending.

Nested PCR is a well-established strategy that significantly enhances detection sensitivity through two rounds of specific amplification. However, the traditional stepwise procedure requires open-tube transfer and dilution of the first-round amplification products, resulting in a cumbersome workflow prone to aerosol contamination that compromises the reliability of test results. To enhance detection sensitivity and specificity while reducing operational steps and contamination risks, our team previously reported a locked nucleic acid (LNA)-based one-tube nested real-time quantitative PCR (LNA-OTN-q-PCR) as well as a multiplex one-tube nested real-time quantitative PCR (mOTNRT-PCR) utilizing LNA, which demonstrated high analytical sensitivity in both singleplex and multiplex formats [21,22]. This method combines LNA technology with nested PCR (NestPCR), in which the two-step nested PCR is performed consecutively within a sealed tube system. Despite their performance advantages, the complexity of LNA primer design and the manual DNA extraction steps have limited their broader clinical application.

This study aims to establish a multiplex one-tube nested real-time quantitative PCR (mONRT-PCR) method that does not rely on LNA-modified primers. Subsequently, this method will be integrated with a point-of-care testing (POCT) instrument for rapid detection of *H. influenzae*, *L. monocytogenes*, and *C. neoformans* in CSF. The mONRT-PCR-POCT assay is a fully automated, closed-system nucleic acid extraction and detection system suitable for clinical point-of-care use. A unified nucleic acid extraction protocol was applied to all target organisms within the sealed cartridge system. The intended role of this assay is to provide rapid molecular support in suspected infectious meningitis cases while conventional microbiological results are pending. The analytical and clinical performance of this mONRT-PCR-POCT assay was evaluated by comparing its results with those obtained from conventional qPCR.

## 2. Materials and Methods

### 2.1. Sample Collection and Genomic DNA Isolation

**Figure 1:**
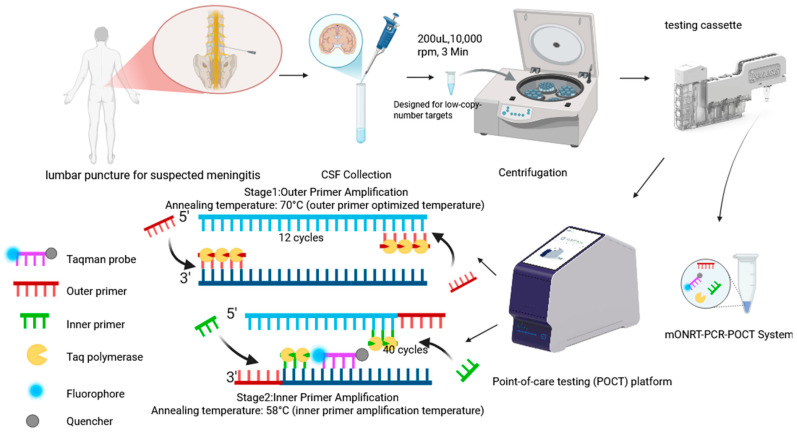
Schematic workflow of the mONRT-PCR-POCT assay for detection of meningitis pathogens from CSF samples. CSF, cerebrospinal fluid; mONRT-PCR, multiplex one-tube nested real-time quantitative PCR; POCT, point-of-care testing.

Standard strains of *H. influenzae* (ATCC 49247), *L. monocytogenes* (ATCC 241656), and *C. neoformans* (ATCC MYA4567) were provided by the Institute of Infectious Diseases, Chinese Center for Disease Control and Prevention (China CDC). Genomic nucleic acids were extracted from these strains using the FastPure^®^ Microbiome DNA Extraction Kit (Vazyme, Nanjing, China) according to the manufacturer’s instructions and stored at −80 °C. The POCT instrument (ENucFlow-PS2 Fully Automated Integrated Nucleic Acid Extraction and Detection System) was provided by WeEasyBio (Shanghai, China). For comparison, the conventional qPCR assay was also performed on the ENucFlow-PS2 platform under the same automated extraction and amplification conditions as the mONRT-PCR-POCT assay. All steps, from nucleic acid extraction to real-time fluorescence PCR detection, were completed automatically within the closed system (Figure 1). A total of 43 clinical residual CSF samples were included in this study, comprising 21 samples positive for *C. neoformans*, 1 for *H. influenzae*, and 1 for *L. monocytogenes*, while the remaining 20 samples were culture-negative and served as negative controls. All CSF specimens included in this study were residual clinical samples collected after routine diagnostic testing in patients undergoing evaluation for suspected central nervous system infections. All samples were processed in the clinical microbiology laboratory of Hebei Provincial People’s Hospital following standard diagnostic procedures. Briefly, CSF specimens were subjected to broth enrichment culture, followed by subculture onto solid agar plates after incubation. Pathogen identification was performed using routine hospital-based methods. Culture-confirmed positive samples, including *C. neoformans*, *H. influenzae*, and *L. monocytogenes*, were used as the reference standard for evaluating assay performance.

### 2.2. Design of ONRT-PCR Primers, Probes, and Plasmid Construction

In nested multiplex PCRs, each target requires two pairs of PCR primers—one pair of inner primers and one pair of outer primers—along with one TaqMan fluorescent probe. Following qPCR primer design principles, sequence alignment was performed using BioEdit software (Ibis Biosciences, Carlsbad, CA, USA). Primer Premier 6 (Premier Biosoft, Palo Alto, CA, USA) and Oligo 7 (Molecular Biology Insights, Cascade, CO, USA) were employed to design outer primers for *H. influenzae*, *L. monocytogenes*, and *C. neoformans*, with their specificity further verified using Primer-BLAST (NCBI, Bethesda, MD, USA) on the National Center for Biotechnology Information (NCBI) website. Primers and probes of the mONRT-PCR assay were developed based on previously published studies targeting the *siaT* gene in *H. influenzae*, the *hly* gene in *L. monocytogenes*, and the *QSP1* gene in *C. neoformans* [23,24,25]. Specifically, the inner primers and probes of the mONRT-PCR assay were cited from published assays with minor modifications, while the outer primers were newly designed in this study. All primer and probe sequences used in this study are listed in Table 1. To evaluate primer and probe suitability, multiple sequence alignment was performed using representative sequences retrieved from the NCBI database. The results confirmed that primer and probe binding regions were highly conserved across analyzed strains, with no mismatches observed at the critical 3′ ends. Detailed alignment results with annotated inner primers, outer primers, and probe regions are provided in Supplementary Figure S1. The annealing temperature of the outer primers was optimized to be 10 °C higher than that of the inner primers to avoid interference by excessively low temperatures or formation of stable primer dimer formation at excessively high temperatures. All primers and probes were synthesized and purified by BiOligo Biotechnology (Shanghai, China). The conserved gene fragments of the three pathogens were cloned into the pUC57 vector respectively, and the recombinant plasmids were synthesized by TsingKe Biotech Corp (Beijing, China). Recombinant plasmid concentrations were quantified using the Qubit^®^ Double-Stranded DNA High Sensitivity Assay Kit (Thermo Fisher Scientific, Waltham, MA, USA) in conjunction with the Qubit 2.0 Fluorometer (Life Technologies, Carlsbad, CA, USA). The calculation method for recombinant plasmid copy number is as follows: Plasmid copy number (copies/μL) = [6.02 × 10^23^ × plasmid concentration (ng/μL) × 10^−9^]/[plasmid length × 660] [26]. The recombinant plasmid was then serially diluted in 1×TE buffer to concentrations ranging from 10^5^ to 10^0^ copies/μL. The diluted solutions were stored at −20 °C for subsequent use.

### 2.3. Establishment and Optimization of the mONRT-PCR Method

The mONRT-PCR assay employed one pair of inner primers and one pair of outer primers for each target within the nested PCR. Based on our group’s prior research, the inner primer reaction achieved maximum amplification efficiency when the inner-to-outer primer final concentration ratio was set at 10:1 and the annealing temperature difference between the two primer pairs exceeded 10 °C [21,22]. The primers used in mONRT-PCR-POCT were identical to those in mONRT-PCR. PCR primers and probes were optimized through screening to ensure efficient amplification of all targets. During the first stage of nested PCR, amplification was primarily driven by the outer primers, allowing rapid amplification of large amounts of template DNA within 12 cycles. Due to the higher annealing temperature at this stage, the inner primers did not anneal effectively and no fluorescence signal was generated. In the second stage, the outer-primer amplicons served as templates for real-time PCR, while the outer primers no longer participated in the reaction and therefore did not interfere with fluorescence detection.

The final composition of the mONRT-PCR system (20 μL) included: 10 μL premix enzyme (Mix); 0.4 μL of each inner forward and reverse primer (10 μmol/L) for the three targets; 0.4 μL of each outer forward and reverse primer (1 μmol/L); 0.2 μL *H. influenzae* probe (10 μmol/L); 0.175 μL *L. monocytogenes* probe (10 μmol/L); 0.3 μL *C. neoformans* probe (10 μmol/L); 1 μL DNA template; and DEPC-treated water (DEPC, diethyl pyrocarboxylate, an RNase-degrading reagent) to a final volume of 20 μL. The amplification program is as follows: Pre-denaturation at 95 °C for 5 min. Stage 1: Reaction at 95 °C for 15 s, followed by reaction at 70 °C for 40 s (12 cycles, outer primer amplification). Second stage: 15 s at 95 °C, 40 s at 58 °C (40 cycles, inner primer amplification). Throughout the detection process, nuclease-free water served as the negative control, while recombinant plasmid and standard bacterial strain nucleic acids diluted to 10^0^ copies/μL were used as positive controls.

For comparison, a conventional multiplex qPCR assay was performed using the same inner primer–probe sets under identical reaction conditions, excluding the outer primers and the initial pre-amplification stage. Conventional qPCR assays were performed on an ARCHIME 916-1006 real-time PCR system (RocGene Technology Limited, Beijing, China) according to standard laboratory procedures. The final composition of the conventional qPCR reaction system (20 μL) was as follows: forward and reverse primers for each of the three pathogens (10 μmol/L), 0.4 μL each; probes (10 μmol/L), 0.2 μL for *H. influenzae*, 0.175 μL for *L. monocytogenes*, and 0.3 μL for *C. neoformans*; 1 μL of DNA template; and DEPC-treated water added to a final volume of 20 μL. The primer and probe sets used in the qPCR assay were identical to the inner primer–probe sets applied in the mONRT-PCR assay. The qPCR assay was established based on previously published sequences and was used as a methodological comparison rather than a routine clinical diagnostic test, and was not validated against commercial kits. The amplification program was as follows: Pre-denaturation at 95 °C for 5 min, followed by 40 cycles of 95 °C for 15 s, and 58 °C for 40 s.

The primers and probes used in the mONRT-PCR-POCT assay were identical to those used in the conventional mONRT-PCR system. The total reaction volume was 25 μL, consisting of 20 μL reaction mixture and 5 μL extracted nucleic acid template. Primer and probe volumes remained unchanged compared with the conventional system. For each experiment, 400 μL of quantified CSF simulant sample was subjected to fully automated nucleic acid extraction in the POCT device. Subsequently, 5 μL of the extracted nucleic acid was automatically transferred into the amplification chamber to complete the nested PCR process in an integrated manner.

### 2.4. Sensitivity and Specificity Assessment

The sensitivity of mONRT-PCR was evaluated using recombinant plasmids containing *H. influenzae*, *L. monocytogenes*, and *C. neoformans* in 10-fold serial dilutions, covering a concentration range from 10^0^ to 10^5^copies/μL. Diluted plasmids were used as templates for mONRT-PCR detection, with DEPC-treated water serving as a negative control. Additionally, genomic DNA was extracted from *H. influenzae*, *L. monocytogenes*, and *C. neoformans* standard strains following the protocol provided by the FastPure^®^ Microbiome DNA Extraction Kit (Vazyme, Nanjing, China). The extracted DNA was subsequently diluted to concentrations ranging from 10^−7^ to 10^−1^ng/μL and subjected to the aforementioned sensitivity testing. Genomic DNA concentrations are expressed as mass input (ng/μL) rather than calculated copy number. The limit of detection was defined as the lowest concentration of target nucleic acids at which 95% of replicates (20 replicates) are detected. Because 1 μL of template DNA was added to each 20 μL PCR, the reported concentrations (copies/μL or ng/μL) correspond directly to the approximate template amount per reaction. To comprehensively evaluate assay performance and minimize potential matrix-related bias, sensitivity testing was conducted in a stepwise manner using recombinant plasmids, purified genomic DNA, and CSF-based simulated samples.

To evaluate analytical specificity and potential cross-reactivity, mONRT-PCR was tested against a panel of common microbial DNA strains encountered in clinical microbiology laboratories. Beyond the target pathogens (*H. influenzae*, *L. monocytogenes*, and *C. neoformans*), the detection scope encompassed 17 other common microorganisms, including *Candida albicans*, *Candida tropicalis*, *Candida glabrata*, *Candida krusei*, *Candida parapsilosis*, *Aspergillus flavus*, *Staphylococcus aureus*, *Klebsiella pneumoniae*, *Streptococcus pneumoniae*, *Pseudomonas aeruginosa*, *Escherichia coli*, *Enterococcus faecalis*, *Enterococcus faecium*, *Enterobacter cloacae*, *Staphylococcus epidermidis*, *Stenotrophomonas maltophilia* and *Mycobacterium tuberculosis* (Supplementary Table S1). Reference strains with ATCC accession numbers were obtained from certified culture collections, and other strains used for specificity validation were sourced from the strain repository maintained by our laboratory, including one clinically isolated strain. This panel was selected to assess assay cross-reactivity with available clinical isolates rather than to represent a comprehensive etiological spectrum of meningitis pathogens.

### 2.5. Preparation of Simulated CSF Samples

Cultures of *H. influenzae*, *L. monocytogenes*, and *C. neoformans* were used to prepare simulated CSF specimens to determine the detection limits of the mONRT-PCR and mONRT-PCR-POCT assays. *H. influenzae* and *L. monocytogenes* were streaked onto antibiotic-free chocolate agar and Columbia blood agar (Tianwei Teda Technology, Beijing, China), respectively, whereas *C. neoformans* was streaked onto Sabouraud agar (Tianjing Sha Gene Technology, Beijing, China). These inoculated strains were then placed in a 5% CO_2_ incubator (Thermo Fisher Scientific, Waltham, MA, USA) and cultured at 37 °C for 24–48 h to allow colony formation. Single colonies were selected from the media for further culture. *H. influenzae* and *L. monocytogenes* were cultured in Brain Heart Infusion (BHI) broth. For *H. influenzae*, the medium was supplemented with X factor (hemin) and V factor (nicotinamide adenine dinucleotide, NAD) according to standard growth requirements. *C. neoformans* was cultured in Yeast Extract-Peptone-Dextrose (YPD) broth. Cultures were incubated separately at 37 °C for *H. influenzae* and *L. monocytogenes* and 27 °C for *C. neoformans* in a shaking incubator (THZ-032, Shanghai Boqi Biotechnology, Shanghai) at 220 rpm overnight. On the following day, 1 mL of each bacterial suspension was collected, centrifuged, and the supernatant was discarded. Pellets of *H. influenzae* and *L. monocytogenes* were washed three times with phosphate-buffered saline (PBS). Ten-fold serial dilutions were prepared in PBS [27], and 10 μL aliquots of each dilution were plated onto Columbia blood agar and antibiotic-free chocolate agar plates. After 24 h of static incubation, colony-forming units (CFU) were counted and analyzed. For *C. neoformans*, the fungal suspension concentration was determined using a hemocytometer. Quantified microbial suspensions were diluted with PBS and added to 1 mL of CSF to generate simulated samples with final concentrations of 5, 10, 20, 50, 80, 100, 200, 500, and 1000 CFU/mL. Simultaneously, control samples were prepared by quantitatively adding the microbial suspension to 1 mL of PBS. Both CSF and PBS samples were incubated at 37 °C under identical conditions to ensure accurate determination of CFU concentrations in the simulated CSF samples.

### 2.6. Evaluation and Comparison of mONRT-PCR-POCT and qPCR Detection Methods in Clinical Specimens

This study evaluated and compared the capabilities of mONRT-PCR-POCT and qPCR in detecting clinical samples using the fully automated closed nucleic acid extraction and detection system (ENucFlow-PS2). A total of 11 CSF specimens with positive *C. neoformans* culture results were collected from Hebei Provincial People’s Hospital for POCT. An additional 20 CSF culture-negative specimens were used as controls. To ensure consistency, nucleic acids extracted fully automatically by the POCT instrument were selected as templates for both mONRT-PCR-POCT and qPCR methods. The detection results obtained by the two methods were subsequently compared to assess their concordance and to comprehensively evaluate their clinical diagnostic performance.

### 2.7. Statistical Analysis

All statistical analyses were performed using SPSS 27.0 software (IBM, Armonk, NY, USA). Agreement between the mONRT-PCR-POCT and qPCR methods was assessed using Cohen’s kappa analysis. A *p*-value < 0.05 was considered to indicate statistical significance.

## 3. Results

### 3.1. Outer Primer Temperature Screening

The annealing temperature of the outer primers is a critical factor influencing amplification efficiency in the nested PCR step of the mONRT-PCR assay. Temperature gradient PCR was performed to screen the optimal annealing temperatures for the outer primers, using paired inner and outer primers targeting each gene. A temperature gradient ranging from 52 °C to 74 °C was programmed, and temperatures from 54 °C to 74 °C were included in the final analysis. For each temperature point, two parallel reactions were conducted: one containing only the outer primers and the target gene, and another containing only the inner primers and the target gene. All reactions within each temperature group were performed under identical conditions. The final products were analyzed by 3.5% agarose gel electrophoresis. The optimal annealing temperature for the outer primers was defined as the temperature at which amplification was observed exclusively with the outer primers, while no amplification occurred with the inner primers. The absence of amplification with the inner primers at the selected temperature ensured that outer primer amplification occurred specifically during the first stage, thereby minimizing potential interference between primer sets and maintaining high amplification efficiency. As shown in Supplementary Figure S2, *H. influenzae* exhibits specific amplification without nonspecific inner primer products at outer primer annealing temperatures of 70–72 °C. *L. monocytogenes* shows the same behavior at 68–70 °C, while *C. neoformans* demonstrates this characteristic at 70–72 °C. Based on these results, 70 °C was selected as the optimal annealing temperature for the outer primers in subsequent mONRT-PCR.

### 3.2. Sensitivity Evaluation of mONRT-PCR and qPCR Detection

To evaluate the analytical sensitivity of the mONRT-PCR assay, recombinant plasmids containing the target sequences of *H. influenzae*, *L. monocytogenes*, and *C. neoformans* at concentrations ranging from 10^5^ to 10^0^ copies/μL, as well as genomic DNA extracted from standard strains and serially diluted from 10^−1^ to 10^−7^ ng/μL, were used as templates. DEPC-treated water served as the negative control in all experiments. As shown in the figure, a Cq value of 37.84 was defined as the positivity cut-off based on the limit of detection (LOD) evaluation using serial dilutions of recombinant plasmid standards and was applied uniformly throughout the study. The LODs were 10^0^ copies/μL for *H. influenzae* and *L. monocytogenes* in the mONRT-PCR method, and 10^1^ copies/μL for *C. neoformans* (Supplementary Figure S3a–c). When genomic DNA was used as template, the LODs were 10^−7^ ng/μL for *H. influenzae* and *L. monocytogenes*, and 10^−6^ ng/μL for *C. neoformans* (Supplementary Figure S4a–c). For comparison, in the qPCR assay, detectable amplification using recombinant plasmids as templates was observed at 10^1^ copies/μL for *H. influenzae* and *L. monocytogenes*, and 10^2^ copies/μL for *C. neoformans* (Supplementary Figure S3d–f). When genomic DNA served as the template, detectable amplification occurred at 10^−7^ ng/μL for *H. influenzae*, 10^−6^ ng/μL for *L. monocytogenes*, and 10^−5^ ng/μL for *C. neoformans* (Supplementary Figure S4d–f). Sensitivity evaluations indicated that mONRT-PCR showed improved detection performance compared with qPCR at lower template concentrations under the same experimental conditions.

### 3.3. Optimization of the mONRT-PCR System

The second amplification stage of mONRT-PCR, in which the first-round amplification products served as templates for the inner-primer qPCR reaction, shared the same buffer system as conventional qPCR (inner-primer qPCR system). Considered the differing optimal annealing temperatures for the target gene in qPCR reactions. The recombinant plasmids with *H. influenzae*, *L. monocytogenes*, and *C. neoformans* concentration of 10^4^ copies/μL were selected as the target gene. By comparing amplification efficiencies across different annealing temperatures in the inner-primer multiplex qPCR system, 58 °C was identified as the optimal annealing temperature for both mONRT-PCR and qPCR (Supplementary Figure S5). Furthermore, given that *H. influenzae* and *L. monocytogenes* exhibited higher sensitivity than *C. neoformans* in qPCR detection, using the 10^5^ to 10^2^ copies/μL *C. neoformans* recombinant plasmid template, the ratios of primers and probes in the triple qPCR system were optimized to ensure high amplification efficiency for all targets. Under the optimized conditions, identical primer concentrations were used for all three targets. The final probe concentrations, achieved by adjusting the added probe volumes from stock solutions of equal concentration (10 μmol/L), were set at 0.2, 0.175, and 0.3 μM for *H. influenzae*, *L. monocytogenes*, and *C. neoformans*, respectively, ensuring efficient and balanced amplification in the mONRT-PCR system (Supplementary Figure S6).

### 3.4. Comparison of mONRT-PCR and qPCR Sensitivity in Simulated Samples

**Figure 2:**
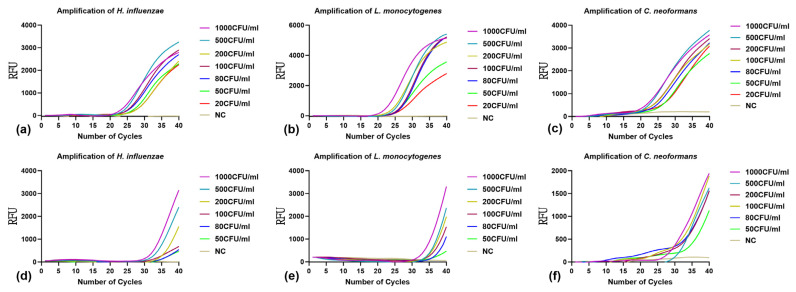
Comparison of mONRT-PCR and qPCR sensitivity for the detection of *H. influenzae*, *L. monocytogenes*, and *C. neoformans* in simulated CSF Samples. The detection limits of mONRT-PCR and qPCR for the three pathogens were evaluated using manually extracted nucleic acids from simulated CSF samples with concentrations ranging from 20 to 1000 CFU/mL. (**a**–**c**) mONRT-PCR results after manual extraction; (**d**–**f**) qPCR results after manual extraction. RFU, relative fluorescence units; NC, negative control; *H. influenzae*, *Haemophilus influenzae*; *L. monocytogenes*, *Listeria monocytogenes*; *C. neoformans*, *Cryptococcus neoformans*; mONRT-PCR, multiplex one-tube nested real-time quantitative PCR; qPCR, quantitative polymerase chain reaction; POCT, point-of-care testing.

mONRT-PCR and qPCR were performed on nucleic acids extracted from simulated CSF samples spiked with *H. influenzae*, *L. monocytogenes*, and *C. neoformans* at concentrations ranging from 20 to 1000 CFU/mL to evaluate the performance of mONRT-PCR for CSF detection. The results are shown in Figure 2. The LOD of mONRT-PCR was 20 CFU/mL for all three pathogens (Figure 2a–c). In contrast, the LOD of qPCR was 50 CFU/mL under the same experimental conditions (Figure 2d–f). The sensitivity evaluation using simulated CSF samples indicated that mONRT-PCR enabled detection at lower bacterial or fungal concentrations compared with qPCR under the same experimental conditions.

### 3.5. Comparative Analysis of mONRT-PCR-POCT and qPCR-POCT

**Figure 3:**
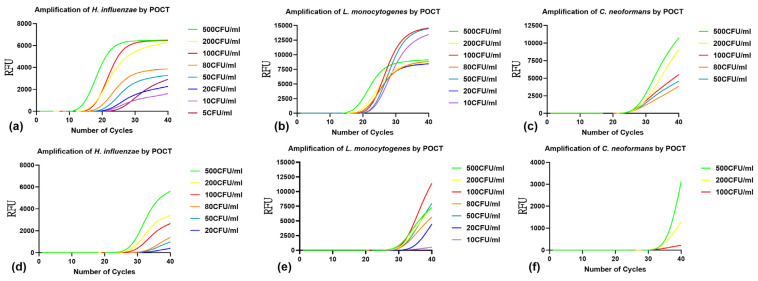
Comparison of mONRT-PCR and qPCR sensitivity for the detection of *H. influenzae*, *L. monocytogenes*, and *C. neoformans* in simulated CSF samples. Nucleic acids were extracted using a fully automated POCT instrument (sample concentration: 5–500 CFU/mL), and the detection limits of mONRT-PCR-POCT and qPCR-POCT for the three pathogens were evaluated. (**a**–**c**) mONRT-PCR results after fully automated POCT extraction; (**d**–**f**) qPCR results after fully automated POCT extraction. RFU, relative fluorescence units; NC, negative control; *H. influenzae*, *Haemophilus influenzae*; *L. monocytogenes*, *Listeria monocytogenes*; *C. neoformans*, *Cryptococcus neoformans*; mONRT-PCR, multiplex one-tube nested real-time fluorescent quantitative PCR; qPCR, quantitative polymerase chain reaction; POCT, point-of-care testing.

Genomic DNA was extracted from simulated samples of the three target pathogens, with concentrations ranging from 5 to 500 CFU/mL. These samples were subsequently analyzed using multiplex qPCR-POCT and mONRT-PCR-POCT on the ENucFlow-PS2 instrument, with results shown in Figure 3. The mONRT-PCR-POCT achieved a limit of detection (LOD) of 5 CFU/mL for *H. influenzae*, 10 CFU/mL for *L. monocytogenes*, and 50 CFU/mL for *C. neoformans*. In contrast, qPCR-POCT produced detectable amplification signals at concentrations of 20 CFU/mL for *H. influenzae* and *L. monocytogenes*, and 200 CFU/mL for *C. neoformans*, under the applied Cq cut-off criteria (as shown in Figure 3).

### 3.6. Evaluation of mONRT-PCR Specificity

In specificity testing, the mONRT-PCR assay developed in this study produced distinct amplification curves only for the target pathogens *H. influenzae*, *L. monocytogenes*, and *C. neoformans*, demonstrating high specificity for simultaneous detection of the target genes. No cross-reactivity was observed with genomic DNA from other microbial pathogens. Strains used in this study are detailed in Supplementary Table S1.

### 3.7. Comparative Evaluation of mONRT-PCR and qPCR Detection Methods in Clinical Samples

A total of 43 CSF clinical samples were analyzed, including 23 positive samples (21 *C. neoformans*, 1 *H. influenzae*, and 1 *L. monocytogenes*) and 20 culture-negative samples. Each sample was tested using both conventional qPCR and mONRT-PCR-POCT, with nucleic acid extracted automatically by the POCT instrument and used as the template for both methods. Among the 23 positive samples, 20 were positive by both mONRT-PCR-POCT and qPCR, while 3 *C. neoformans*-positive samples were negative by qPCR but positive by mONRT-PCR-POCT. All 20 culture-negative samples were negative by both methods. No cases were observed in which qPCR yielded a positive result while mONRT-PCR-POCT was negative. The quantification cycle (Cq) values for the 18 qPCR-positive *C. neoformans* samples ranged from 33.87 to 37.59. The Cq values for the *H. influenzae* and *L. monocytogenes* positive samples were 35.90 and 36.33, respectively. The Cohen’s kappa value was 0.861 (*p* < 0.001) indicating almost perfect agreement between the two methods. The detection results for each pathogen are summarized in Table 2.

## 4. Discussion

### 4.1. Comparison with Existing Multiplex PCR-Based POCT Systems

POCT has gained widespread application due to its advantages of short turnaround time, proximity to the patient, and ease of operation by non-specialized personnel [28]. Commercial multiplex PCR-based POCT systems, such as the FilmArray and QIAstat-Dx meningitis panels [18], enable rapid and comprehensive screening of CSF pathogens. To date, comprehensive analytical sensitivity data for these systems remain limited in the peer-reviewed literature. It should be noted that commercial meningitis panels are designed to simultaneously detect a broad range of pathogens within a single assay, which inherently increases assay complexity and may affect performance under certain clinical conditions. Several clinical evaluations have noted that sensitivity may vary depending on target organism and specimen type [19]. Therefore, differences in assay design should be considered when interpreting LoD comparisons, and such comparisons should be interpreted with caution due to variations in target number and experimental conditions. Reported analytical sensitivities of commercial multiplex meningitis panels vary depending on platform and strain [29,30]. According to manufacturers’ instructions, the FilmArray ME Panel demonstrates LoDs of approximately 10^3^ CFU/mL for *H. influenzae* and *L. monocytogenes*, and 10^2^ CFU/mL for *C. neoformans*. Similarly, the QIAstat-Dx ME Panel reports LoDs ranging from 10^2^ to 10^3^ CFU/mL for *H. influenzae*, 10^3^ to 10^4^ CFU/mL for *L. monocytogenes*, and approximately 10^2^ to 10^3^ CFU/mL for *C. neoformans*. Within the context of these design differences, the mONRT-PCR-POCT assay in this study achieved LoDs of 5, 10, and 50 CFU/mL for *H. influenzae*, *L. monocytogenes*, and *C. neoformans*, respectively, suggesting that the proposed method provides a relatively high level of analytical sensitivity within a streamlined multiplex design. Due to the lack of access to commercial multiplex panels during the study period, a direct head-to-head comparison could not be performed; however, the observed performance suggests that the proposed method operates within a comparable analytical range. In contrast to highly multiplexed commercial panels, the present assay focuses on a limited number of targets with a simplified design, which may reduce inter-target competition and potentially improve performance in low-pathogen-load samples. Importantly, this assay enables simultaneous detection of multiple pathogens within a fully integrated and automated platform, while inherently involving trade-offs between analytical sensitivity and assay complexity.

The inclusion of multiple targets within a single reaction may introduce competition among targets and reagents, which could potentially affect performance in low-pathogen-burden samples, a scenario commonly encountered in CSF, particularly following partial antimicrobial treatment [30]. This limitation may be relevant for pathogens such as *H. influenzae* and *L. monocytogenes*, as well as for *C. neoformans*, where analytical sensitivity may be influenced by fungal burden [31]. Despite these constraints, the mONRT-PCR-POCT method enabled detectable amplification at lower template concentrations compared with conventional qPCR under the same experimental conditions, Notably, *C. neoformans* was additionally identified in three samples by mONRT-PCR-POCT but negative by qPCR. These additional detections were consistent with culture-based identification results, suggesting a potential advantage in detecting low-abundance targets, although this observation should be interpreted cautiously due to the limited sample size.

### 4.2. Analytical Performance and Methodological Considerations of the mONRT-PCR-POCT Assay

The mONRT-PCR-POCT assay combines the rapid detection capability of POCT with the enhanced sensitivity of one-tube nested PCR [32,33]. Specific nested primers and TaqMan probes were designed to target conserved gene sequences of each pathogen. By avoiding the use of costly and difficult-to-design LNA probes and instead employing simple, unmodified primers, the detection panel can be more readily updated and expanded according to clinical needs or regional epidemiological characteristics, thereby improving flexibility. This design enables rapid and sensitive pathogen detection with a total turnaround time of 1 h and 47 min. Isothermal amplification methods such as LAMP and RPA have also been explored for point-of-care applications due to their ability to amplify nucleic acids under constant temperature conditions without thermal cycling. However, certain isothermal approaches, particularly LAMP, require relatively complex primer design and optimization, which may complicate assay development and limit performance in multiplexed settings [34,35]. In contrast, the current mONRT-PCR-POCT strategy is based on a conventional PCR framework combined with nested amplification, allowing more straightforward primer and probe design within a well-established real-time PCR format. The workflow is simplified, as nucleic acid extraction and two rounds of nested PCR amplification are integrated within a single cartridge. The integration of nucleic acid extraction and amplification into a fully automated closed platform reduces contamination risk and improves workflow standardization [36]. The assay also requires only a small CSF volume (200–400 μL), which may reduce patient discomfort associated with repeated sampling.

### 4.3. Sample Processing Strategy for CSF Specimens

Prior to testing CSF specimens, centrifugal concentration and collection were selected in this study as the pretreatment for nucleic acid extraction. CSF represents a diagnostic sample with low biological load and low matrix complexity. In low-biological-load specimens such as CSF, synovial fluid, and pleural or ascites fluid, the types and concentrations of chemical components and biomolecules (e.g., proteins, lipids, cellular debris) are relatively simple, with minimal background interference. Compared to blood—a sample characterized by high biological load, high matrix complexity, and strong background interference—this inherent simplicity of matrix composition allows certain methods requiring specific enrichment steps in blood (e.g., enrichment using recombinant human mannose-binding lectin magnetic beads (M1 beads)) [37], may be replaced or supplemented by simpler, faster physical methods (e.g., centrifugation) in CSF. Given the low biological load and simple matrix composition of CSF, centrifugal concentration represents a cost-effective and widely accepted enrichment strategy that can improve detection sensitivity [38,39,40]. This provides stable, reliable technical assurance for detecting low-concentration samples while reducing detection errors caused by uneven pathogen distribution or random sampling.

### 4.4. Limitations and Future Perspectives

However, the mONRT-PCR-POCT detection method has certain limitations. First, the efficiency of fungi lysis requires further improvement. The current ENucFlow-PS2 system employs a co-processed nucleic acid extraction protocol for both bacteria and fungi within a fully sealed cartridge, which limits the flexibility to optimize lysis conditions during processing. Consequently, despite incorporating ultrasonic disruption, the capsular structure of *C. neoformans* may not be fully disrupted, potentially reducing nucleic acid release efficiency and contributing to a relatively higher limit of detection for this pathogen. Potential mitigation strategies include programmatically extending magnetic bead agitation duration, increasing ultrasonic disruption power, and optimizing nucleic acid lysis buffer conditions. In addition, polysaccharide components of *C. neoformans* have been reported to interfere with PCR amplification [41], and further optimization strategies may help mitigate this effect. Second, the current mONRT-PCR assay is limited to qualitative detection and does not provide quantitative information on nucleic acid levels. In addition, a direct comparison with commercial panels was not performed. Finally, the clinical validation of this study is limited by both sample size and pathogen distribution. A total of 23 positive samples were included, comprising 21 *C. neoformans*, with only one case each of *H. influenzae* and *L. monocytogenes*. Therefore, the clinical applicability of the assay for non-cryptococcal targets should be interpreted with caution and considered preliminary. This limitation is associated with the availability of well-characterized CSF specimens under the study conditions. In contrast, the relatively larger number of *C. neoformans*–positive samples allowed a more informative assessment for this pathogen. Future studies with larger, multicenter cohorts and a more balanced distribution of pathogens will be essential to further validate the diagnostic performance and clinical utility of this platform.

## 5. Conclusions

Despite these limitations, the mONRT-PCR-POCT assay developed in this study provides a rapid and sensitive approach for the detection of *H. influenzae*, *L. monocytogenes*, and *C. neoformans* in CSF samples. By integrating multiplex one-tube nested PCR with a fully automated POCT platform, the assay enables streamlined and closed-system detection, which may be advantageous for early diagnosis of meningitis-associated pathogens. Further optimization of the platform, including integration with more compact and portable devices and reduction in reaction time, may help to improve its practicality for clinical application.

## Figures and Tables

**Table 1:** The mONRT-PCR primer and probe sequences.

Species	Type	Sequence	Source
*H. influenzae*	HI-mONRT-PCR-OF	TCGGTCATTACACAGGAGGAATGGGACACGTTAATATCGG	This study
	HI-mONRT-PCR-OR	CCAAAAAGATTGTTTAAAGCTGCTGCAAAGTTGTTCTCTCGT	This study
	HI-qPCR-F	AATGCGTGATGCTGGTTATGAC	[23]
	HI-qPCR-R	AAGAGTTTTGCGATAGATTCATTGG	[23]
	HI-P ^a^	GGAGGAATTACTGCTGCTTCTTGT	This study
*L. monocytogenes*	LM-mONRT-PCR-OF	AGTCTACCAATTGCGCAACAAACTGAAGCAAAGGA	This study
	LM-mONRT-PCR-OR	GTAACCTTTTCTTGGCGGCACATTTGTCACTGCAT	This study
	LM-qPCR-F	TTTCATCCATGGCACCACC	[24]
	LM-qPCR-R	ATCCGCGTGTTTCTTTTCGA	[24]
	LM-P ^b^	CGCCTGCAAGTCCTAAGACG	[24]
*C. neoformans*	CN-mONRT-PCR-OF	CACCAACACCCAAGTCTATCTTCAAAGCCTCACAC	This study
	CN-mONRT-PCR-OR	ATGAGGACGAGGTCAAGTAGAACACGGATAGGAGA	This study
	CN-qPCR-F	ACCACTCTTTTCACTGCTG	[25]
	CN-qPCR-R	GGCGCCGAAGTTGTTAG	[25]
	CN-P ^c^	CTTGTCCTCATCGCCCCGGCCCTC	[25]

Note: ^a^ Probe modifications: FAM 6-carboxyfluorescein, BHQ1 black hole quencher 1, ^b^ Probe modifications: VIC 6-phosphoramidite, BHQ1 black hole quencher 1, ^c^ Probe modifications: ROX Carboxy-X-rhodamine, BHQ2 black hole quencher 2, *H. influenzae*, *Haemophilus influenzae*; *L. monocytogenes*, *Listeria monocytogenes*; *C. neoformans*, *Cryptococcus neoformans*; qPCR: quantitative polymerase chain reaction, mONRT-PCR: Multiplex one-step nested real time polymerase chain reaction.

**Table 2:** Detection of mONRT-PCR-POCT and qPCR in clinical samples.

Species	mONRT-PCR-POCT		qPCR	
	Positive	Negative	Positive	Negative
*C. neoformans*	21	22	18	25
*H. influenzae*	1	42	1	42
*L. monocytogenes*	1	42	1	42

Note: Kappa value was calculated based on overall paired results across all clinical samples. *H. influenzae*, *Haemophilus influenzae*; *L. monocytogenes*, *Listeria monocytogenes*; *C. neoformans*, *Cryptococcus neoformans*; mONRT-PCR, multiplex one-tube nested real-time fluorescent quantitative PCR; qPCR, quantitative polymerase chain reaction; POCT, point-of-care testing.

## Data Availability

The original contributions presented in the study are included in the article/Supplementary Materials, further inquiries can be directed to the corresponding authors.

## Supplementary Materials

The following supporting information can be downloaded at: https://www.mdpi.com/article/10.3390/pathogens15050456/s1, Figure S1: To further evaluate the conservation and suitability of the primer and probe binding regions, multiple sequence alignment analyses were performed using representative sequences retrieved from the NCBI database; Figure S2: Optimization of outer primer annealing temperature conditions; Figure S3: Sensitivity analysis of mONRT-PCR and qPCR using recombinant plasmids; Figure S4: Sensitivity analysis of mONRT-PCR and qPCR using genomic DNA from reference strains; Figure S5: Optimization of annealing temperature for the inner-primer multiplex qPCR system using *H. influenzae*, *L. monocytogenes*, and *C. neoformans* recombinant plasmids at 10^4^ copies/μL; Figure S6: Optimization of primer–probe ratios in the multiplex qPCR system using *C. neoformans* recombinant plasmids at concentrations ranging from 10^5^ to 10^2^ copies/μL; Table S1: Microbial strains used in specificity assay.
