# Supplementary material for: A Multiplex One-Tube Nested Real-Time PCR Assay for the Point-of-Care Testing of Infectious Meningitis
Source: Pathogens. 2026 Apr 22;15(5):456. doi: 10.3390/pathogens15050456 (PMC13209304; doi:10.3390/pathogens15050456)
Supplement: Supplementary file 1 [file pathogens-15-00456-s001.zip › pathogens-4266448-supplementary.pdf]

Supplementary Materials

Supplementary Figure S1

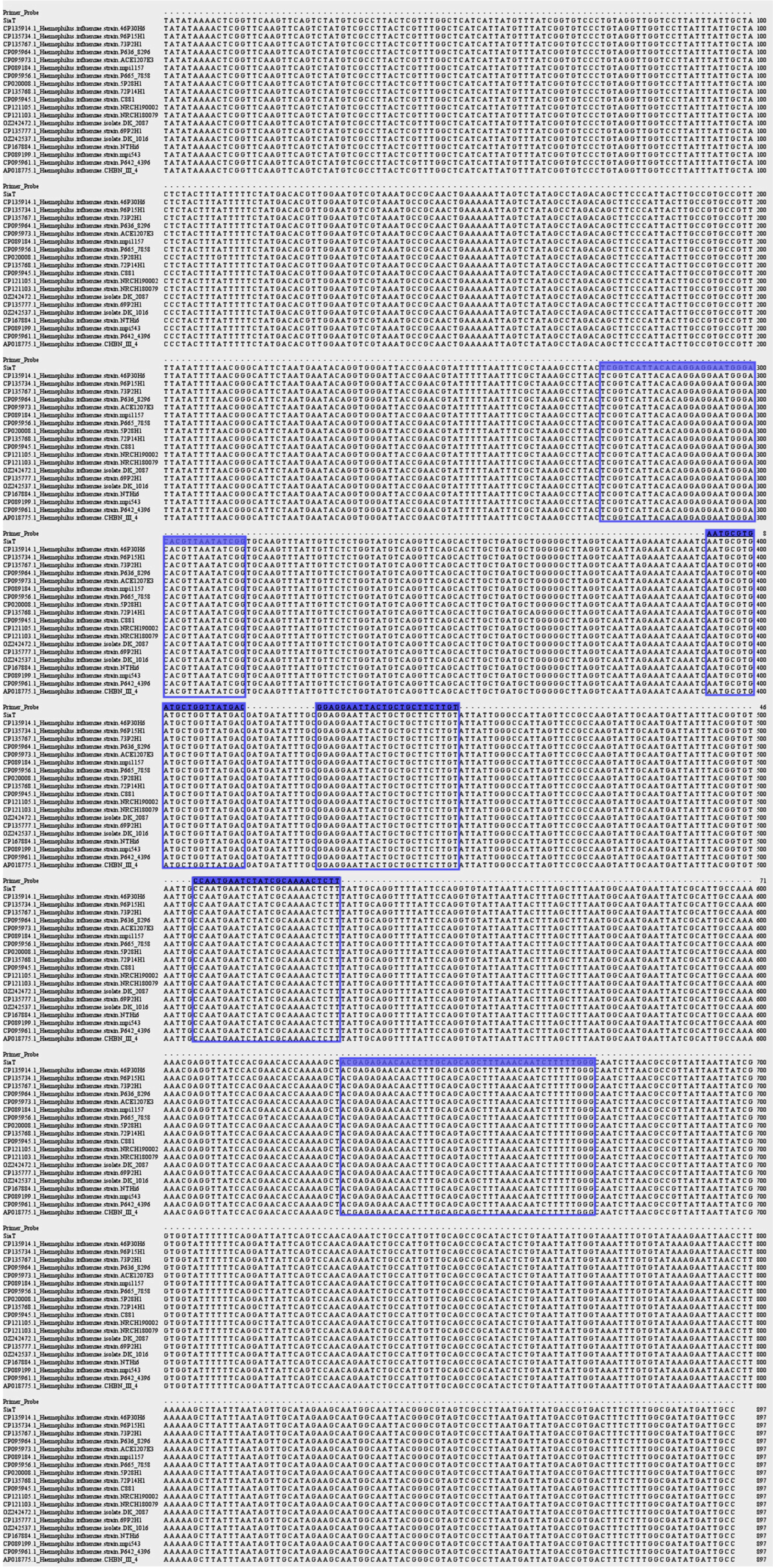

(a)

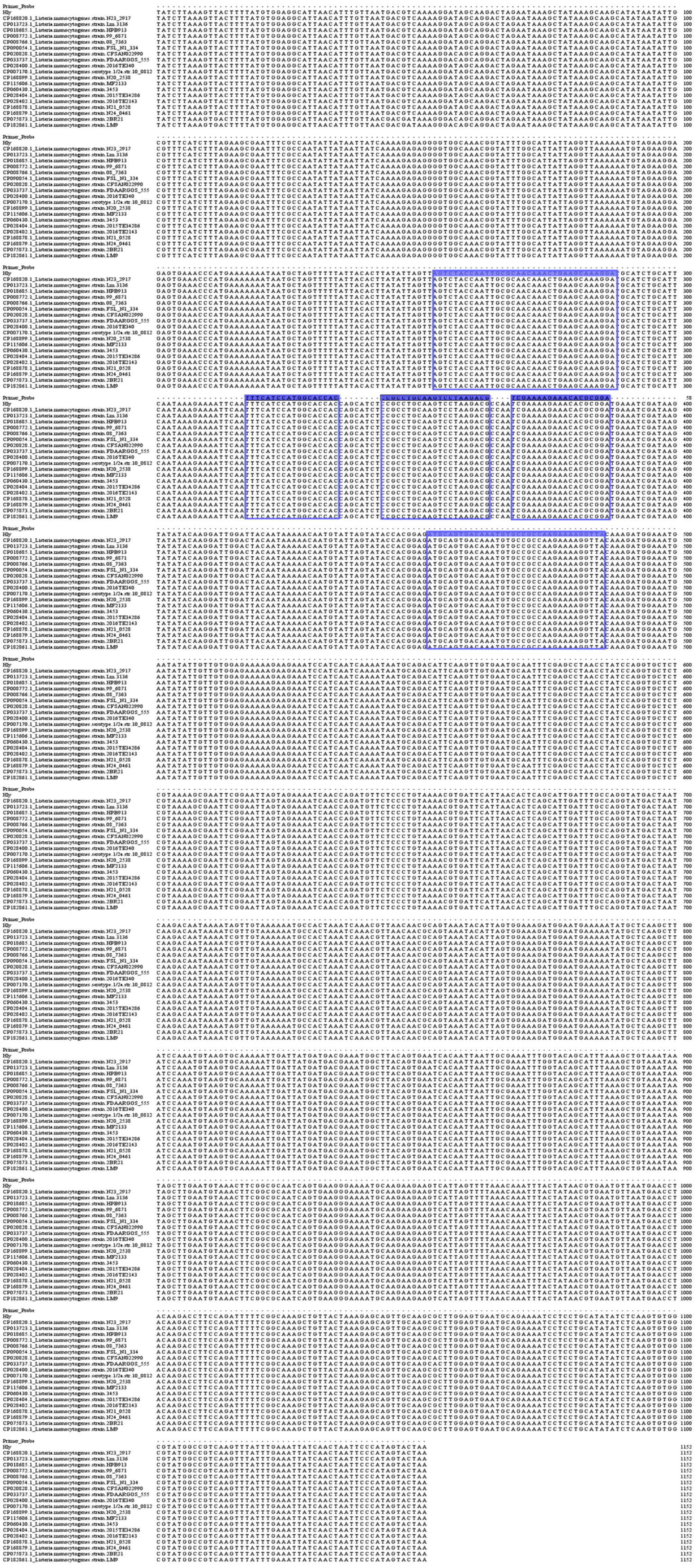

(b)

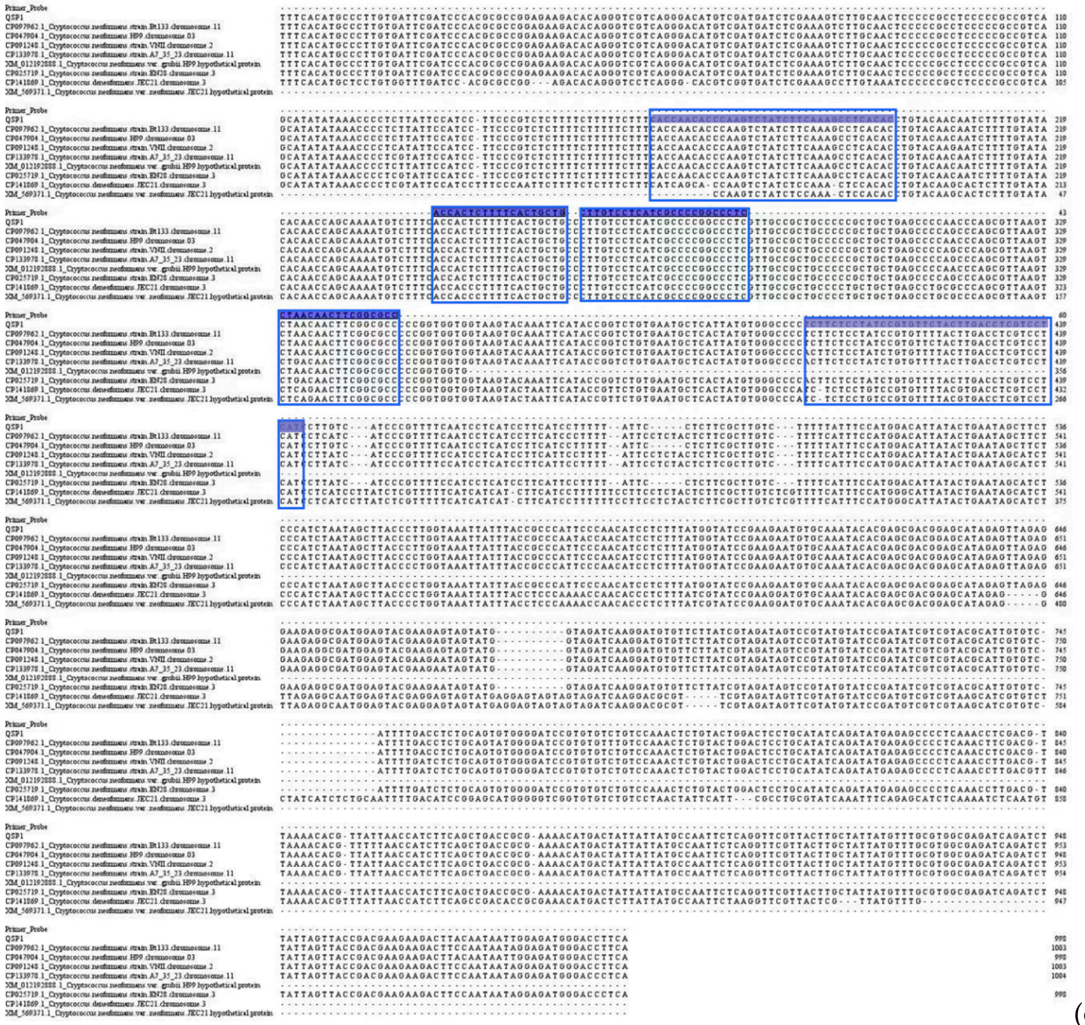

(c)

Supplementary Figure S1: To further evaluate the conservation and suitability of the primer and probe binding regions, multiple sequence alignment analyses were performed using representative sequences retrieved from the NCBI database. For the bacterial targets, a total of 20 representative sequences were included for each gene (*siaT* for *H. influenzae* and *hly* for *L. monocytogenes*), covering diverse strains. The alignment results demonstrated that all primer and probe binding regions, including both inner and outer primers, were fully conserved across all analyzed sequences, with 100% sequence identity and no mismatches observed. For the fungal target (*QSP1* gene of *C. neoformans*), 9 representative sequences were included. The alignment revealed that the primer and probe binding regions remained conserved across all sequences, with no mismatches identified at the critical 3′ ends of the primers. Limited sequence variation, including occasional mismatches or gaps, was observed only in internal regions of the amplicon outside the primer and probe binding sites. These variations are unlikely to affect amplification efficiency or assay performance. The inner primers and probes used in this study were derived from previously published studies, while the outer primers were newly designed. The positions of all primers and probes are annotated in the alignment figures. These results support the specificity and robustness of the primer and probe design across representative strains.

(a) Multiple sequence alignment of the *siaT* gene of *H. influenzae*. The binding regions of outer

primers, inner primers, and probe are indicated. The alignment demonstrates complete conservation across 20 representative strains, with 100% sequence identity and no mismatches observed within all primer and probe binding regions.

(b) Multiple sequence alignment of the *hly* gene of *L. monocytogenes*. The binding regions of outer primers, inner primers, and probe are indicated. The alignment shows full conservation across 20 representative strains, with no mismatches observed in any primer or probe binding regions.

(c) Multiple sequence alignment of the *QSP1* gene of *C. neoformans*. The binding regions of outer primers, inner primers, and probe are indicated. The alignment demonstrates conserved primer and probe binding regions across 9 representative sequences. No mismatches were observed at the critical 3′ ends of the primers. Minor sequence variations, including occasional mismatches or gaps, were observed only in internal regions outside the primer and probe binding sites.

H. influenzae, *Haemophilus influenzae*; *L. monocytogenes*, *Listeria monocytogenes*; *C. neoformans*, *Cryptococcus neoformans*

Supplementary Figure S2

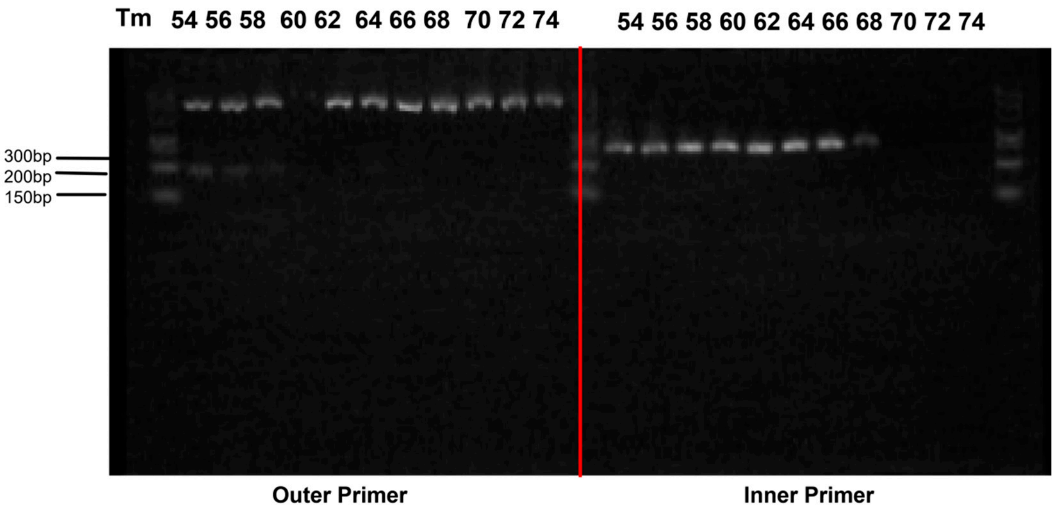

(a)

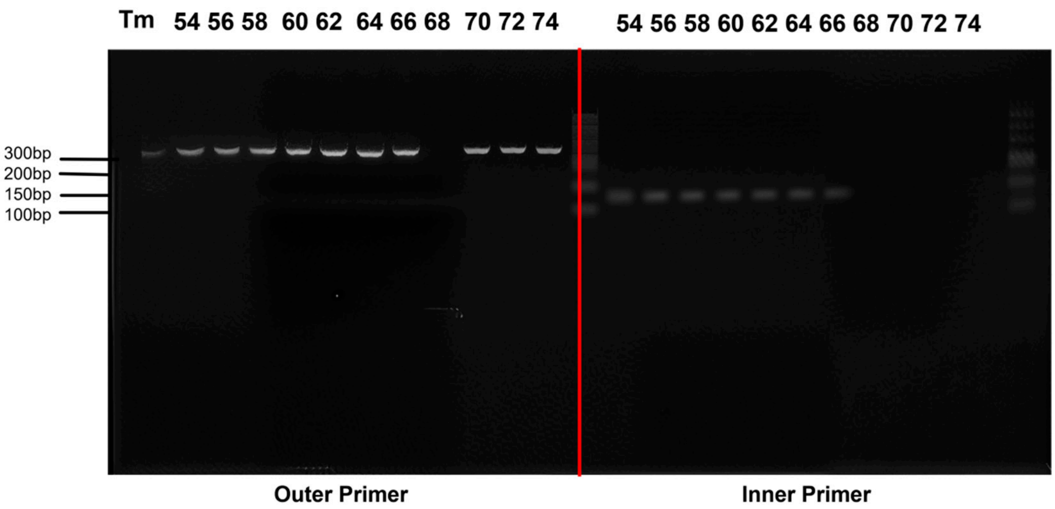

(b)

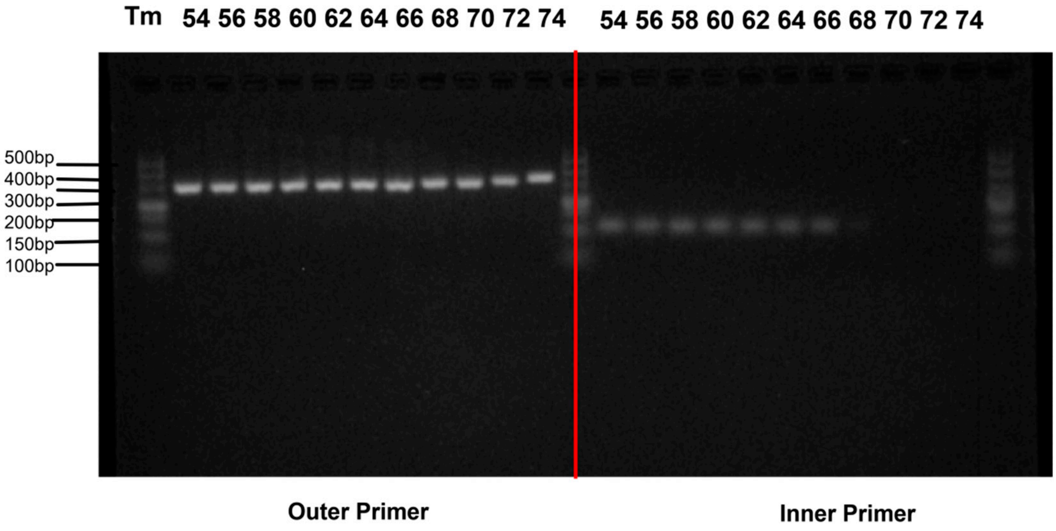

(c)

Supplementary Figure S2: Optimization of outer primer annealing temperature conditions. (a) Optimization of the outer primer annealing temperature for *H. influenzae*, with gel electrophoresis images obtained using outer and inner primers at different temperatures (54–74 °C). (b) Optimization of the outer primer annealing temperature for *L. monocytogenes*, with gel electrophoresis images obtained using outer and inner primers at different temperatures (54–74 °C). (c) Optimization of the outer primer annealing temperature for *C. neoformans*, with gel electrophoresis images obtained using outer and inner primers at different temperatures (54–74 °C). In each panel, the left lanes show amplification products generated using outer primers, while the right lanes show amplification products generated using inner primers. *H. influenzae*, *Haemophilus influenzae*; *L. monocytogenes*, *Listeria monocytogenes*; *C. neoformans*, *Cryptococcus neoformans.*

Supplementary Figure S3

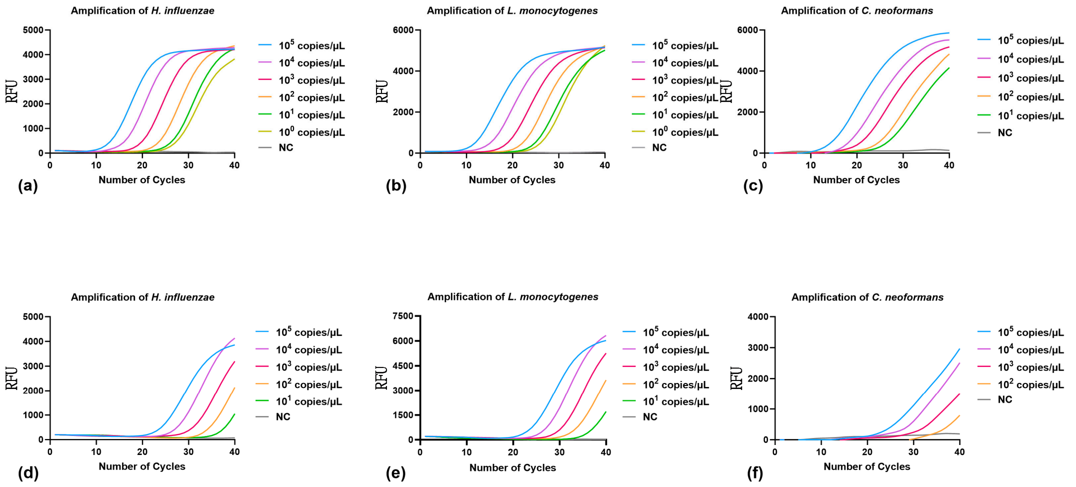

Supplementary Figure S3: Sensitivity analysis of mONRT-PCR and qPCR using recombinant plasmids. The LOD of mONRT-PCR and qPCR were evaluated using recombinant plasmids containing the target sequence of *H. influenzae*, *L. monocytogenes*, and *C. neoformans*. RFU: Relative Fluorescence Units; NC: Negative Control; *H. influenzae*, *Haemophilus influenzae*; *L. monocytogenes*, *Listeria monocytogenes*; *C. neoformans*, *Cryptococcus neoformans*; mONRT-PCR, multiplex one-tube nested real-time fluorescent quantitative PCR; qPCR, quantitative polymerase chain reaction.

Supplementary Figure S4

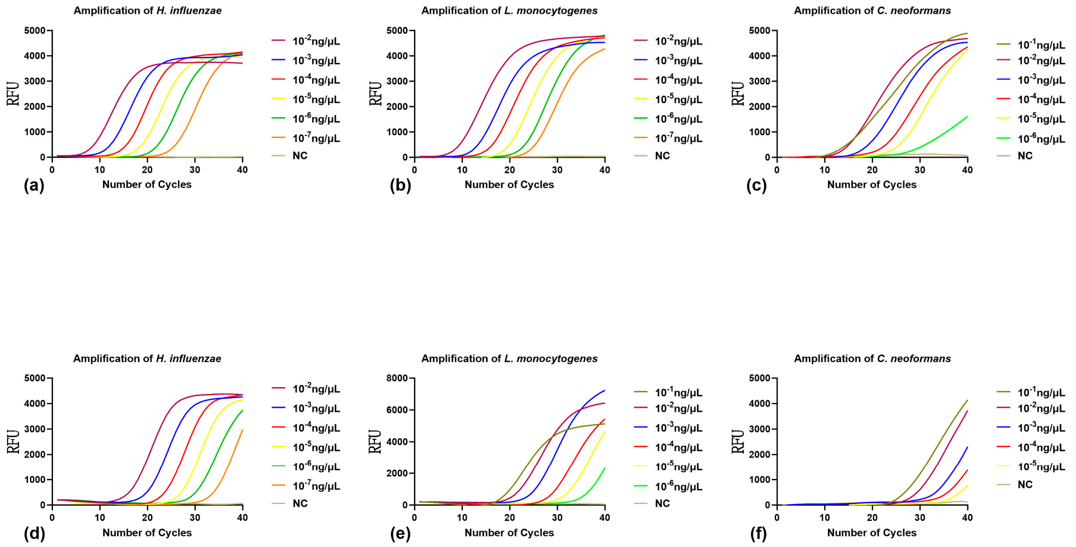

Supplementary Figure S4: Sensitivity analysis of mONRT-PCR and qPCR using genomic DNA from reference strains. The LOD of mONRT-PCR and qPCR were evaluated using genomic DNA extracted from reference strains of *H. influenzae*, *L. monocytogenes*, and *C. neoformans*. RFU: Relative Fluorescence Units; NC: Negative Control; *H. influenzae*, *Haemophilus influenzae*; *L. monocytogenes*, *Listeria monocytogenes*; *C. neoformans*, *Cryptococcus neoformans*; mONRT-PCR, multiplex one-tube nested real-time fluorescent quantitative PCR; qPCR, quantitative polymerase chain reaction.

Supplementary Figure S5

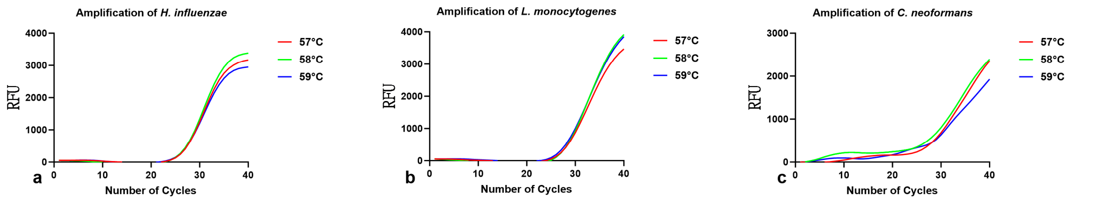

Supplementary Figure S5: Optimization of annealing temperature for the inner-primer multiplex qPCR system using *H. influenzae*, *L. monocytogenes*, and *C. neoformans* recombinant plasmids at 10^4 copies/μL. (a) Inner-primer multiplex qPCR amplification curves for *H. influenzae* at different annealing temperatures (57, 58, and 59 °C).(b) Inner-primer multiplex qPCR amplification curves for *L. monocytogenes* at different annealing temperatures (57, 58, and 59 °C).(c) Inner-primer multiplex qPCR amplification curves for *C. neoformans* at different annealing temperatures (57, 58, and 59 °C). *H. influenzae*, *Haemophilus influenzae*; *L. monocytogenes*, *Listeria monocytogenes*; *C. neoformans*, *Cryptococcus neoformans*; qPCR, quantitative polymerase chain reaction.

Supplementary Figure S6:

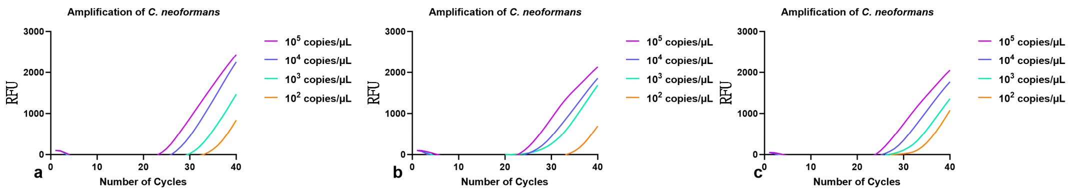

Supplementary Figure S6: Optimization of primer–probe ratios in the multiplex qPCR system using *C. neoformans* recombinant plasmids at concentrations ranging from 10^5 to 10^2 copies/μL. (a) Multiplex qPCR amplification curves obtained using identical primer volumes (0.4 μL) and a probe ratio of 2:1.75:3 for *H. influenzae*, *L. monocytogenes*, and *C. neoformans*, respectively.(b) Multiplex qPCR amplification curves obtained with constant primer concentrations for *H. influenzae* and *L. monocytogenes*, while adjusting the *C. neoformans* primer (0.3 μL) and probe (0.2 μL) volumes.(c) Multiplex qPCR amplification curves obtained using identical primer volumes (0.4 μL) and a probe ratio of 2:1.75:2 for *H. influenzae*, L. *monocytogenes*, and *C. neoformans*. *H. influenzae*, *Haemophilus influenzae*; *L. monocytogenes*, *Listeria monocytogenes*; *C. neoformans*, *Cryptococcus neoformans*;qPCR, quantitative polymerase chain reaction.

Supplementary Table S1

Supplementary Table S1 Microbial strains used in specificity assay

Strains	Origin	*H. influenzae*	*L. monocytogenes*	*C. neoformans*
Haemophilus influenzae	ATCC 49247	Positive	Negative	Negative
Listeria monocytogenes	ATCC 241656	Negative	Positive	Negative
Cryptococcus neoformans	ATCC MYA4567	Negative	Negative	Positive
Candida albicans	ATCC 753	Negative	Negative	Negative
Candida tropicalis	ATCC 750	Negative	Negative	Negative
Candida glabrata	ATCC 2001	Negative	Negative	Negative
Candida krusei	ATCC 6258	Negative	Negative	Negative
Candida parapsilosis	ATCC 22019	Negative	Negative	Negative
Aspergillus flavus	Isolated strains	Negative	Negative	Negative
Staphylococcus aureus	ATCC 29213	Negative	Negative	Negative
Klebsiella pneumoniae	ATCC 11296	Negative	Negative	Negative
Pseudomonas aeruginosa	ATCC29213	Negative	Negative	Negative
Escherichia coli	ATCC 25922	Negative	Negative	Negative
Staphylococcus epidermidis	ATCC 35984	Negative	Negative	Negative
Enterococcus faecium	ATCC 19434	Negative	Negative	Negative
Enterococcus faecalis	ATCC 29212	Negative	Negative	Negative
Enterobacter cloacae	ATCC 13047	Negative	Negative	Negative
Streptococcus pneumoniae	ATCC 6303	Negative	Negative	Negative
Mycobacterium tuberculosis	ATCC 25177	Negative	Negative	Negative
Stenotrophomonas maltophilia	Isolated strains	Negative	Negative	Negative

Note： *H. influenzae, Haemophilus influenzae; L. monocytogenes, Listeria monocytogenes; C. neoformans, Cryptococcus neoformans.*
